# Quantum modelling and molecular docking evaluation of some selected quinoline derivatives as anti-tubercular agents

**DOI:** 10.1016/j.heliyon.2020.e03639

**Published:** 2020-03-31

**Authors:** Shola Elijah Adeniji, Gideon Adamu Shallangwa, David Ebuka Arthur, Mustapha Abdullahi, A.Y. Mahmoud, Abdurrashid Haruna

**Affiliations:** Chemistry Department, Ahmadu Bello University, Zaria Nigeria

**Keywords:** Pharmaceutical chemistry, Theoretical chemistry, Molecular docking, Quinoline, QSAR, Tuberculosis

## Abstract

*Mycobacterium tuberculosis* has instigated a serious challenge toward the effective treatment of tuberculosis. The reoccurrence of the resistant strains of the disease to accessible drugs/medications has mandate for the development of more effective anti-tubercular agents with efficient activities. Time expended and costs in discovering and synthesizing new hypothetical drugs with improved biological activity have been a major challenge toward the treatment of multi-drug resistance strain *M. tuberculosis* (TB). Meanwhile, to solve the problem stated, a new approach i.e. QSAR which establish connection between novel drugs with a better biological against *M. tuberculosis* is adopted*.* The anti-tubercular model established in this study to forecast the biological activities of some anti-tubercular compounds selected and to design new hypothetical drugs is subjective to the molecular descriptors; AATS7s, VE2_Dzi, SpMin7-Bhe and RDF110i. The significant of the model were observed with R^2^ of 0.8738, R^2^ adj of 0.8351 Q_cvˆ2 of 0.7127 which served as criteria to substantiate the QSAR model. More also, the model significant with the QSAR external validation criterial ‘‘(R^2^test) of 0.7532. Ligand-receptor interactions between quinoline derivatives and the receptor (DNA gyrase) was carried out using molecular docking technique by employing the PyRx virtual screening software and discovery studio visualizer software. Furthermore, docking study indicates that compounds 10 of the derivatives with promising biological activity have the utmost binding energy of -18.8 kcal/mol. Meanwhile, the interaction of the standard drug; isoniazid with the target enzyme was observed with the binding energy -14.6 kcal/mol which was significantly lesser than the binding energy of the ligand (compound 10). This implies that ligand 10 could be used as a structural template to design better hypothetical anti-tubercular drugs with more efficient activities. The presumption of this research aid the medicinal chemists and pharmacist to design and synthesis a novel drug candidate against the tuberculosis. Moreover, in-*vitro* and in-*vivo* test could be carried out to validate the computational results.

## 1 Introduction

Over the years, tuberculosis has been a serious threat to mankind which is caused by *M. tuberculosis*. World Health Organization (2018), has reported cases of 9.0 million infected people, 360,000 HIV patient whom were leaving with tuberculosis, death of 230,000 children and death of 1.6 million people worldwide [1]. Some of the notable commercial sold drugs administered to people infected with tuberculosis are isoniazide (INH), pyrazinamide (PZA), rifampicin (RMP) and para-amino salicylic acid (PAS). The emergence of multi-drug resistance strain of *M.TB* toward the aforementioned medications has steered to advances in searching for new and better approach that is precise and fast in developing a novel compound with improved biological activity against *M. tuberculosis.*

For the time being, extensively used computational method i.e. QSAR is a theoretical approach in designing and predicting new hypothetical drug candidate [2]. Multi-variant QSAR model is expressed mathematically to relates the biological activity of each compound with its respective molecular structures.

Class of substituted quinoline has been reported to as an anti-tubercular agents [3]. The derivaties of this class have demonstrated efficient and promising anti-tubercular activities against the strain of multidrug resistance tuberculosis. Nonappearance of resistance with known tuberculosis drugs designated that ring-substituted quinolone derivaties perhaps act by different mechanism which is more efficient than the currently drugs. Consequently, substituted quinoline is promising and considered as an essential and novel class of anti-tuberculois drugs.

Meanwhile, some prominent researchers [4, 5, 6, 7] have successful established QSAR models to show the relationship between some anti-*M. tuberculosis* inhibitor's such as; chalcone, quinoline, 7-methylquinolone, pyrrole and their respective biological activities using QSAR approach. Nevertheless, report has shown that docking study and QSAR to explain the relationship and interaction between the compound and the target is yet to be established. Hence, this research was aimed to evaluate the ligand-receptor complex formed via docking approaching and to build a robust QSAR model with high predictability to predict the activities against *M-tuberculosis* via in silico method.

## 2 Material and method

### 2.1 Collection of data set

The molecules comprising the dataset of quinoline reported as a potential compounds against *M.TB* used in this study was obtained in the literature [3]. Forty derivatives of quinoline were collected while twenty 27 derivatives with good anti-*M. tuberculosis* were selected for the modelling study. The list of the compounds were presented in Table 1.

**Table 1:** Molecular structures of inhibitory compounds and their derivatives as anti-tubercular agents.

S/N	Molecular structure	Observed Activity (%)	Observed Activity (pA)	Calculated Activity (pA)	Residual
1^t^	(E)-2-(2-(4-methoxybenzylidene)hydrazinyl)-N-phenylquinoline-4-carboxamide	99	9.4858	9.7207	-0.2349
2	(E)-2-(2-(4-methoxybenzylidene)hydrazinyl)-N-phenylquinoline-4-carboxamide	14	6.9651	6.8856	0.0795
3^t^	(E)-N-benzyl-2-(2-(pyridin-3-ylmethylene)hydrazinyl)quinoline-4-carboxamide	23	7.2487	6.4992	0.7495
4	(E)-N-benzyl-2-(2-(furan-2-ylmethylene)hydrazinyl)quinoline-4-carboxamide	20	7.1586	6.9618	0.1968
5^t^	(E)-N-benzyl-2-(2-(thiophen-2-ylmethylene)hydrazinyl)quinoline-4-carboxamide	30	9.4639	9.7549	-0.2910
6	(E)-2-(2-(anthracen-9-ylmethylene)hydrazinyl)-N-benzylquinoline-4-carboxamide	20	6.9432	6.9198	0.0234
7^t^	(E)-N-benzyl-2-(2-((4-methoxynaphthalen-1-yl)methylene)hydrazinyl)quinoline-4-carboxamide	16	7.2268	6.5334	0.6934
8^t^	(E)-N-benzyl-2-(2-(2-methylpropylidene)hydrazinyl)quinoline-4-carboxamide	42	7.1367	6.9960	0.1407
9^t^	(E)-N-benzyl-2-(2-propylidenehydrazinyl)quinoline-4-carboxamide	27	7.3893	7.1755	0.2138
10	(E)-N-benzyl-2-(2-benzylidenehydrazinyl)quinoline-4-carboxamide	99	7.2498	7.0087	0.2411
11	(E)-N-benzyl-2-(2-(4-methoxybenzylidene)hydrazinyl)quinoline-4-carboxamide	21	7.1132	7.7017	-0.5885
12	(E)-N-(5-phenylpentyl)-2-(2-(pyridin-4-ylmethylene)hydrazinyl)quinoline-4-carboxamide	30	7.5695	7.7356	-0.1661
13	(E)-2-(2-(furan-2-ylmethylene)hydrazinyl)-N-(5-phenylpentyl)quinoline-4-carboxamide	15	7.2598	6.5187	0.7411
14	(E)-N-(5-phenylpentyl)-2-(2-(thiophen-2-ylmethylene)hydrazinyl)quinoline-4-carboxamide	21	9.575	9.6508	-0.0758
15^t^	(Z)-2-(2-(anthracen-9-ylmethylene)hydrazinyl)-N-(5-phenylpentyl)quinoline-4-carboxamide	23	7.229	7.9095	-0.6805
16	(E)-2-(2-((4-methoxynaphthalen-1-yl)methylene)hydrazinyl)-N-(5-phenylpentyl)quinoline-4-carboxamide	40	7.4432	7.4348	0.0084
17	(E)-2-(2-(2-methylpropylidene)hydrazinyl)-N-(5-phenylpentyl)quinoline-4-carboxamide	42	7.0467	7.1958	-0.1491
18	(E)-2-(2-benzylidenehydrazinyl)-N-(5-phenylpentyl)quinoline-4-carboxamide	21	7.2407	7.2472	-0.0065
19	(E)-2-(2-(4-methoxybenzylidene)hydrazinyl)-N-(5-phenylpentyl)quinoline-4-carboxamide	40	7.3751	7.6971	-0.3220
20	(E)-(2-(2-(4-methoxybenzylidene)hydrazinyl)quinolin-4-yl)(morpholino)methanone	10	7.7072	7.3417	0.3655
21	(E)-(4-methylpiperazin-1-yl)(2-(2-(pyridin-4-ylmethylene)hydrazinyl)quinolin-4-yl)methanone	28	7.6348	7.2968	0.3380
22	(E)-(2-(2-(furan-2-ylmethylene)hydrazinyl)quinolin-4-yl)(4-methylpiperazin-1-yl)methanone	21	6.2348	6.3486	-0.1138
23^t^	(E)-(2-(2-((4-methoxynaphthalen-1-yl)methylene)hydrazinyl)quinolin-4-yl)(4-methylpiperazin-1-yl)methanone	18	7.663	7.7607	-0.0977
24	(E)-(4-methylpiperazin-1-yl)(2-(2-(2-methylpropylidene)hydrazinyl)quinolin-4-yl)methanone	52	6.8074	6.8325	-0.0251
25	(E)-(2-(2-benzylidenehydrazinyl)quinolin-4-yl)(4-methylpiperazin-1-yl)methanone	9	7.3333	7.3807	-0.0474
26	(E)-(2-(2-(4-methoxybenzylidene)hydrazinyl)quinolin-4-yl)(4-methylpiperazin-1-yl)methanone	30	7.1551	7.4150	-0.2599
27	(E)-N-phenyl-2-(2-(thiophen-2-ylmethylene)hydrazinyl)quinoline-4-carboxamide	26	7.1682	7.5235	-0.3553

### 2.2 Inhibition activities

The activities of the dataset primarily reported in percentage (%) was converted to logarithm scale with the aid of Eq. (1) so as to maintain normal distribution and to increase the linearity value of the activities. The difference between the observed and calculated activities is the residual value reported in Table 1.

(1) [4] $$\text{pBA}=\text{log}\left[\left(\frac{Molecularweight_{\left(g/mol\right)}}{Dose_{\left(g/mol\right)}}\right)\left(\frac{\text{percentage }\left(\text{\%}\right)}{100-\text{percentage }\left(\text{\%}\right)}\right)\right]$$

Key: Superscript **t** denoted the test set. The observed (experimental) activity is gotten from the literature which were reported as percentage (%) inhibition. The calculated activity (pA) is generated using QSAR model built in this study. The residual values is the difference between the observed activity (pA) and calculated activity (pA). Leverage value for each compound represent the diagonal matrix element with which the applicability domain boundary is defined. The chemical structure of each compound is presented in Table S1 in EMS.

### 2.3 Molecular optimization

Spartan 14 software version 1.1.4 was used to optimize all the inhibitory compounds so as achieve a steady conformation at a minimum-energy. Thereafter, the removal of energy strain from the molecules and complete optimization were achieved with the aid of Mechanics Force Field (MMFF) and Density Functional Theory (DFT) [4].

### 2.4 Generation of descriptor

The numeral term based on the association between the biological activity of each molecule and its molecular structure is expressed in term of ‘‘descriptor’’. This was achieved using PaDEL software V.2.20 with a total of 1879 descriptors generated.

### 2.5 Data normalization and pretreatment

QSAR is influenced by each variable (descriptor) in order to generate a good model. Therefore, the descriptors values generated from PaDE software V2.20 were normalized using Eq. (2) so as give the descriptor equal chance at the point inception [8, 9].

(2) $$\text{D}=\frac{d_{1}-d_{min}}{d_{max}-d_{min}}$$

The maximum and minimum value for each descriptors are denoted by dmax and dmin, d_1_ is the descriptor value for each of the molecule. Thereafter, the data normalized were pretreated with pretreatment software (https://dtclab.webs.com/software-tools) so as to remove redundant descriptors.

### 2.6 Generation training and test set

The division of the dataset in a ratio 7:3 i.e. the training and test set was accomplished using the algorithm of Kennard and Stone which was incorporated into DTC lab software. Building of the model and internal validation test were performed on the training set. Meanwhile the confirmation of model of the developed model was performed on test set [9].

### 2.7 Derivation of the model and models and validation

The modelling tool to develop the multi-variant equations by placing the activity data in the last column of Microsoft Excel 2013 spread sheet and the technique to select optimum descriptors form the training set was accomplished using Multi-linear regression Approach (MLR) and Genetic Function Approximation (GFA). The internal validation test to affirm the robustness of the model built and its predictability was also accomplished in Material Studio software V.8.0 and reported.

### 2.8 Evaluation of leverage values (applicability domain)

Influential and outlier molecule present in the dataset were determined by employing the applicability domain approach. Meanwhile, leverage *h*_*i*_ approach as defined in Eq. (3) was used define applicability domain space ±3 for outlier molecule [9].

(3) $$h_{i}=M_{i}{\left(M^{T}M\right)}^{-1}M_{i}^{T}$$

Where *M*_*i*_ represent the matrix of *i* for the training set. *M* represent the n×dmatrix descriptor for the training set, $M^{T}\text{is the transpose of the training set }\left(M\right)$. $M_{i}^{T}$ represent the transpose matrix *M*_*i*_. Meanwhile, the warning leverage h∗ defined in Eq. (4) is the boundary to establish the presence an influential molecule.

(4) $$h*=3\frac{\left(d+1\right)}{N}$$

Where *N* is the total number of training set and *d* is the total number of descriptors present the built model.

### 2.9 Y-randomization validation assessment

Y-Randomization assessment is one of the validation criteria which has to be considered so as to affirm that the model is not built by chance [9, 10]. Random shuffling of the data was executed on training data following the principle laid by [11]. The activity data (dependent variable) were shuffled while the descriptors (independent variables) were kept unchanged in order to generate the Multi-linear regression (MLR) model. For the developed QSAR to pass the Y-Randomization test, the values for *R*^2^ and Q^2^ must be significantly low for numbers of trials while Y-randomization Coefficient $\left(\text{c}R_{p}^{2}\right)$ shown in Eq. (5) must be ≥ 0.5 so as to establish the strength of the model.

(5) $$\text{c}R_{p}^{2}=R\times {\left[R^{2}-{\left(R_{r}\right)}^{2}\right]}^{2}$$

### 2.10 Affirmation of the build model

The criteria for validating both test and training set were reported and compared with the generally accepted threshold value shown in Table 6 for any QSAR model [9, 11, 12, 13] in order to assert the consistency, fitting, stability, strength and predictability of the developed models.

### 2.11 Docking studies

#### 2.11.1 Receptor (DNA gyrase) preparation

**Figure 1:**
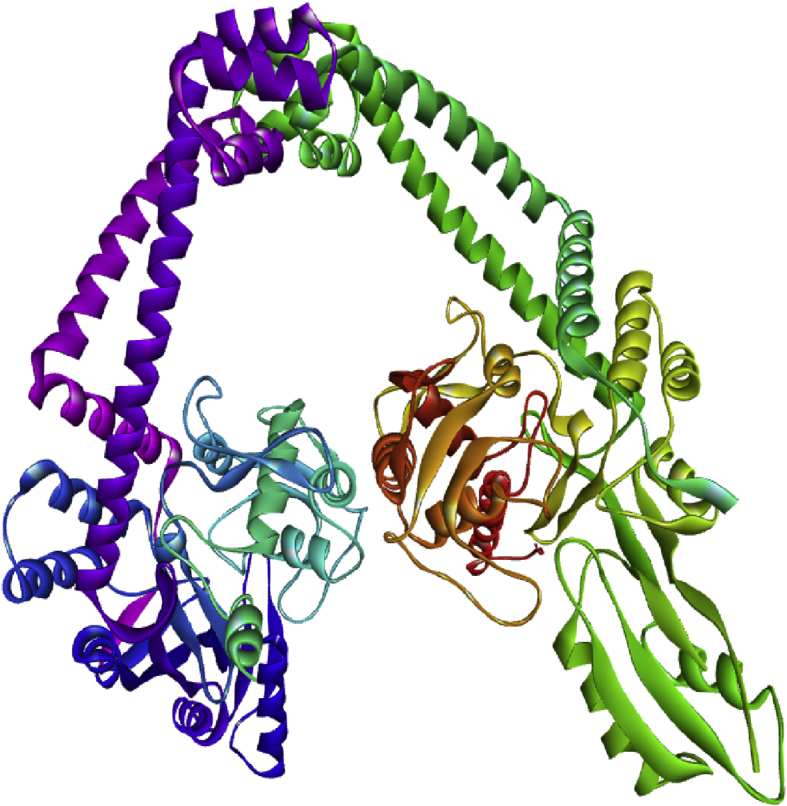
Crystal structure of DNA gyrase.

The DNA gyrase (31FZ) crystal form shown in Figure 1 was downloaded from PDB [15]. All imported foreign matters like cofactors and ligands allied with the enzyme were removed using Discovery Studio Visualizer software. Later on, the target protein was saved format in (PDB) i.e. recommend format for Discovery Studio Visualizer and Pyrx software. Thereafter, the target protein saved in PDB format was imported in the Pyrx software and converted as macro molecules [4, 14].

#### 2.11.2 Ligand preparation

The stable conformation of quinoline derivatives at a minima energy were achieved by employing Spartan software which serve as an optimized tool. The ligands optimized were later saved as a PDB format in order to be recognized by the Pyrx software. Later on, the ligands saved in PDB format were imported in the Pyrx software and converted as micro molecules [4, 14].

#### 2.11.3 Docking of receptor and ligand

Ligand-receptor interactions between quinoline derivatives and the receptor (DNA gyrase) was carried out using molecular docking technique by employing the PyRx virtual screening software. The PyRx software [https://pyrx.sourceforge.io/], is a software used for execution virtual screening. PyRx uses AutoDock Vina and AutoDock 4.2 as docking softwares. Discovery Studio Visualizer software version 2016 was used to visualized and analyzed the docked results [4, 14].

## 3 Results and discussion

### 3.1 Discussion of QSAR studies

Optimum model for forecasting the derivatives of 2, 4-disubstituted quinoline against *M. tuberculosis* was successfully achieved by adopting the combination of computational and theoretical method. Dataset of 27 molecules was partitioned into 19 training data and 8 test data using. The 19 training set compounds were used to derive QSAR model using Multi-linear regression technique which also served as data set for internal validation test while the confirmation of the model was conducted on the test set.

The observed activities reported in literature and the calculated activities calculated for all the anti-tubercular compounds were presented in Table 1. The residual value which is the difference between the observed activity and calculated activity was observed to be significant low. The low residual value designated the predictability of the model.

Optimum (2D and 3D) descriptors that efficiently describe the anti-tubercular compounds in relation to their biological activities selected by GFA approach were reported in Table 2.

**Table 2:** Descriptors used in the model.

S/NO	Descriptors symbols	Name of descriptor(s)	Class
1	AATS7s	Average Broto-Moreau autocorrelation - lag 7/weighted by I-state	2D
2	VE2_Dzi	Average coefficient sum of the last eigenvector from Barysz matrix/weighted by first ionization potential	2D
3	SpMin7-Bhe	Smallest absolute eigenvalue of Burden modified matrix - n 7/weighted by relative Sanderson electronegativities	2D
4	RDF110i	RDF90i is 3D radial distribution function at 2.5 inter-atomic distance weighted by atomic masses.	3D

Various statistical analysis were conducted on the calculated descriptors in order to assess the validity of the descriptors. Evaluation of the VIF (Variance inflation factor) was determined in order to define the degree of correlation between each the descriptor. Generally, VIF value equal to 1 or falls with 1 and 5 signify non-existence of inter-correlation present in each of the descriptors. However, VIF more than 10 signify that the developed model is unsteady hence, the model should be re-checked if necessary. Regarding the VIF for each descriptor which was found to be less than 5 as reported in Table 3 affirm that the descriptors were significantly orthogonal to each order since there is no inter-correlation between them.

**Table 3:** Statistical consideration to validate the descriptors.

Descriptor	Standard regression coefficient $\left(b_{j}\right)$	Mean Effect (ME)	P- Value (Confidence interval)	VIF	Standard Error
AATS7s	-0.4202	-0.4398	1.29E-04	1.3099	-0.0463
VE2_Dzi	0.2128	0.2013	4.22E-02	3.7809	-0.0232
SpMin7-Bhe	-0.695	-0.7142	8.73E-03	1.6582	-0.0481
RDF110i	0.8408	0.8627	6.62E-06	2.1683	-0.0476

The influence each descriptor plays in the built model was estimated by determining the $b_{j}^{s}$ (standard regression coefficient) and ME (mean effect) [9, 16]. The magnitude and signs for $b_{j}^{s}$and ME values reported in Table 3 indicate strength and direction with which each descriptor influence the activity model. The association between the descriptors and the activity of each compound was determined by one way Analysis of variance (ANOVA). The probability value of each of the descriptor at 95% confidence level were found to be (p < 0.05) as presented in Table 3. Therefore this signify that the alternative hypothesis is accepted. This implies that there is a direct connection between the biological activity of each compound and the descriptor swaying the built model. The null hypothesis proposing no direct relationship between biological activity of each compound and the descriptor swaying the built model is rejected. To further justify the validation of the descriptors in the activity model, Pearson correlation statistic was conducted to also check whether there is inter-correlation between each descriptors. The correlation coefficient between each descriptors reported in Table 4 were all <±0.8. Hence this implies that all the descriptors were void of multicollinearity.

**Table 4:** Validation of the descriptors using Pearson's correlation matrix.

	AATS7s	VE2_Dzi	SpMin7-Bhe	RDF110i
AATS7s	1			
VE2_Dzi	0.5120	1		
SpMin7-Bhe	0.0591	0.0173	1	
RDF110i	0.5192	-0.3810	-0.0720	1

Validation results for both the external and internal assessment to assure that model is reliable presented in Table 5. These results affirm the stability and robustness of the model to be valid since the calculated parameters were all in full agreement with general validation criteria presented in Table 5.

**Table 5:** Validation parameters to confirm the built model.

S/NO	Validation Parameters	Formula	Threshold		Model
**Internal Validation**					
1	Friedman LOF	$\frac{SEE}{{\left(1-\frac{C+d\times p}{M}\right)}^{2}}$	Significantly low		0.0476
2	R-squared	$1-\left[\frac{\sum {\left(Y_{exp-Y_{pred}}\right)}^{2}}{\sum {\left(Y_{exp-{\bar{Y}}_{training}}\right)}^{2}}\right]$	${\text{R}}^{2}>0.6$		0.8653
3	Adjusted R-squared	$\frac{R^{2}-P\left(n-1\right)}{n-p+1}$	${\text{R}}_{\text{adj}}^{2}>0.6$		0.8351
4	Cross validated R-squared ($Q_{cv}^{2})$	$1-\left[\frac{\sum {\left(Y_{pred-Y_{exp}}\right)}^{2}}{\sum {\left(Y_{exp-{\bar{Y}}_{training}}\right)}^{2}}\right]$	${\text{Q}}^{2}>0.6$		0.7127
5	Significant Regression				Yes
6	Critical SOR F-value (95%)	$\frac{\sum {\left(Y_{pred-Y_{exp}}\right)}^{2}}{\text{p}}/\frac{\sum {\left(Y_{pred-Y_{exp}}\right)}^{2}}{\text{N}-\text{p}-1}$	${\text{F}}_{\left(\text{test}\right)}>2.09$		2.6296
7	Min expt. error for non-significant LOF (95%)				0.0628

**Model Randomization**					

8	Average of the correlation coefficient for randomized data (${\bar{R}}_{r}$)		$\bar{\text{R}}<0.5$		0.4403
9	Average of determination coefficient for randomized data ( ${\bar{R}}_{r}^{2})$		${\bar{R}}_{r}^{2}<0.5$		0.2723
10	Average of leave one out cross-validated determination coefficient for randomized data ( ${\bar{Q}}_{r}^{2}$ )		${\bar{Q}}_{r}^{2}<0.5$		-1.4310
11	Coefficient for Y-randomization (c$R_{p}^{2})$	${\text{R}}^{2}\times \;\left(1-\sqrt{\left|{\text{R}}^{2}-{\bar{\text{R}}}_{\text{r}}^{2}\right|}\;\right)$	${\text{cR}}_{\text{p}}^{2}>0.6$		0.6703

**External validation**					

12	Slope of the plot of Observed activity against Calculated activity values at zero intercept **(K)**	$\frac{\Delta Y_{\mathrm{Obs}}}{\Delta Y_{\mathrm{cal}}}$	0.85 < k < 1.15	1.016	
13	Slope of the plot of Calculated against Observed activity at zero intercept **(k′)**	$\frac{\Delta Y_{\mathrm{Cal}}}{\Delta Y_{\mathrm{Obs}}}$	0.85 < k < 1.15	0.9210	
16	$/r_{0}^{2}-{r^{'}}_{0}^{2}/$		<0.3	0.0142	
17	$\frac{r^{2}-r_{0}^{2}}{r^{2}}$		<0.1	0.0032	
18	$\frac{r^{2}-{r^{'}}_{0}^{2}}{r^{2}}$		<0.1	0.0421	
19	$R_{test}^{2}$	$1-\;\frac{\sum {\left({\text{Y}}_{\text{ext}}-{\hat{\text{Y}}}_{\text{ext}}\right)}^{2}}{\sum {\left({\text{Y}}_{\text{ext}}-\bar{\text{Y}}\right)}^{2}}$	${\text{R}}_{\text{pred}}^{2}>0.6$	0.7883	

### 3.1.1 Model built

$$\begin{array}{ll} \text{pBA}= & -7.230978576\times \text{AATS}7\text{s}\\ & +0.230874209\times \text{VE}2\text{\_Dzi}\\ & -3.620817054\times \text{SpMin}7-\text{Bhe}\\ & +0.402780284\times \text{RDF}90\text{i}\\ & +8.307195832 \end{array}$$

The coefficient of Y- Randomization $\left(\text{c}R_{p}^{2}\right)$ of 0.6703 greater than threshold value of 0.5 reported in Table 6 provide a reasonable supports that the model built is valid and not just obtained accidental.

**Table 6:** Y- Randomization Parameters test.

**Model**	R	Rˆ2	Qˆ2
Original	0.8593	0.8114	0.7872
Random 1	0.5221	0.2527	-1.0932
Random 2	0.4534	0.2576	-0.2205
Random 3	0.8151	0.4748	0.0005
Random 4	0.5504	0.3201	-0.1104
Random 5	0.3295	0.1141	-0.8591
Random 6	0.6757	0.2425	0.0091
Random 7	0.4217	0.1515	-0.9175
Random 8	0.5121	0.2568	-0.6852
Random 9	0.4236	0.2536	-0.7012
Random 10	0.6726	0.3843	-0.0166

**Random Models Parameters**			
Average r:	0.4403		
Average rˆ2:	0.2723		
Average Qˆ2:	-1.4310		
cRpˆ2:	0.6703		

**Figure 2:**
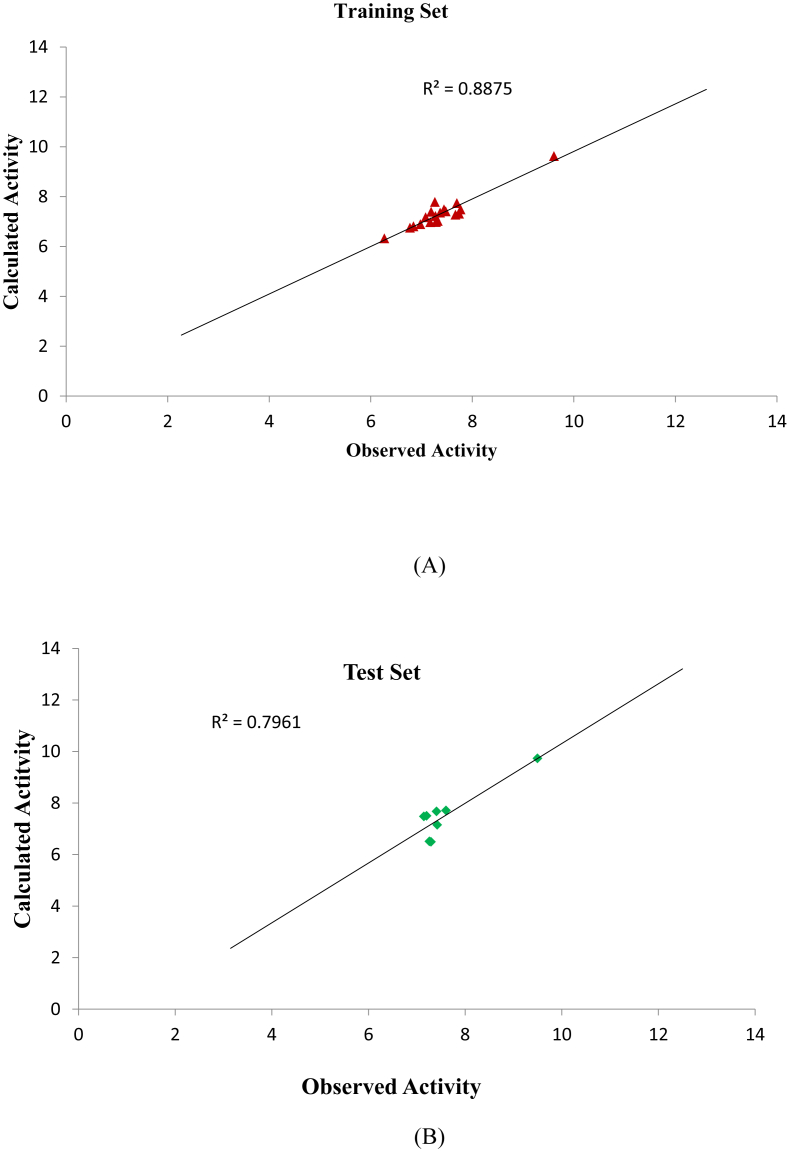
(A) is the plot of calculated activity against observed activity of training set (B) is plot of calculated activity against observed activity of test set.

**Figure 3:**
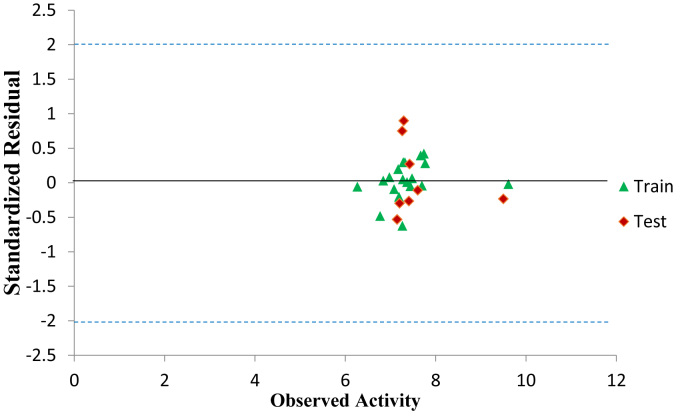
Standardized residual activity versus observed activity.

**Figure 4:**
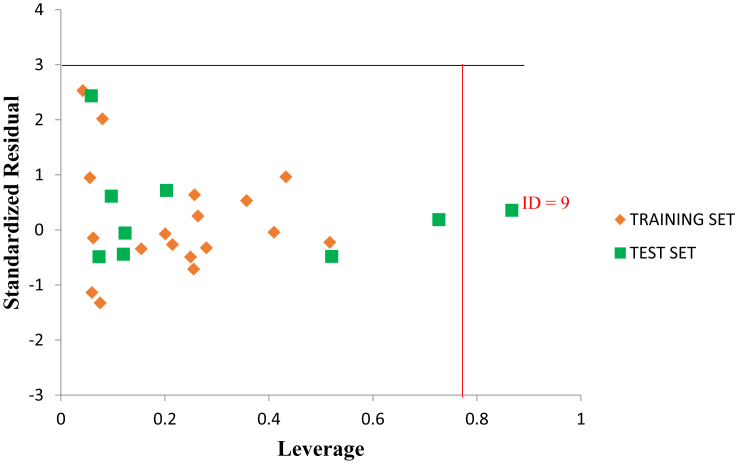
The Williams plot of the standardized residuals versus the leverage value.

The graphical representation to show the degree of correlation between the calculated activities and observed training and test data activities were shown in Figure 2. The R^2^ of 0.8653 and 0.7883 for both the training and test data shows that there is a high correlation existing between the calculated activities and observed activities of the training and test data which were also in line with the established QSAR threshold values reported in Table 5. Indication of computational incompetency and inaccuracy was void in the model derived since all the standard residual values for the dataset were found within the defined boundary of ±2 on the standard residual activity axis shown in Figure 3. The Williams plot to show the Applicability Domain space (AD) is shown in Figure 4. However, the leverage value of compound (number 9) is observed to be higher than the h∗ = 0.79 (i.e. warning leverage). Thus it can be infer that compound (number 9) an influential molecule. Moreover, it is also observed that all the compounds were within the defined space of ±3 which indicates that no compound is said to be outlier.

### 3.1.2 Mechanistic information of descriptors in the model built

**AATS7s** descriptor is an Average Broto-Moreau autocorrelation - lag 7/weighted by I-state auto-correlation. This descriptor is on the basis of longitudinal autocorrelation function which measures the correlation between space separating the (lag) and the molecular or atomic properties. More also, the descriptor is well-stated on the molecular graphs by means of electronegativity (e), mass (m) and inductive effect respectively on the atoms 7 of the molecule. Reference to the established information, it is suggested that distribution of electrons and atomic masses that comprises the molecules had great substantial influence on anti-tubercular activity. The coefficient and the mean effect of this descriptor are negative which indicate that the inhibitory activities of quinoline derivatives will increases with decrease in the descriptor value.

**VE2-Dzi** is Average coefficient sum of the last eigenvector from Barysz matrix/weighted by first ionization potential. The positive mean effect value of these descriptors designates that the activities of quinoline derivatives will increases with decrease in the descriptor which suggest the potency of the compounds against *M. tuberculosis*.

**SpMin7-Bhe** descriptors is among the improved Eigen descriptors. This descriptors is a molecular structure descriptor been formulated from a novel symbol of chemical configuration. The descriptor has a low absolute eigenvalue of Burden reformed matrix/weighted by relative van der Waals size. Coefficient and the mean effect of the descriptor is seen to be negative which proposes that the activity is inversely related to the descriptor. Therefore, the negative sign implies that groups of molecule having more branching diminishes the activity of the active compound toward *M. tuberculosis* as the descriptor increases.

**RDF90i** is among the 3-dimensional radial distribution function at 2.5 inter-atomic distance. This descriptor is independent of the spin and volume of the molecule. This descriptor also give information on the the steric hindrance. Moreover, it provides reasonable information on the planar, ring types, non-planar systems, bond distances and atom types. The influence of this descriptor in the model proposed the existence of connection between the 3-dimensional structure of the molecule and the biological activity against tuberculosis. It obviously that the biological activity of the compound is greatly influenced by the positive mean effect of the descriptor.

### 3.2 Docking studies

#### 3.2.1 Binding energy evaluation in the ligand-receptor complex

Elucidation of binding interactions and the binding mode between the inhibitory compound and target (DNA gyrase) was achieved via molecular docking studies. The QSAR on the anti-tubercular activity of compound 10 correlates coincide with the binding affinity. Therefore, this signify that there is relationship between the QSAR and molecular docking results at (p < 0.05). Ligand (compounds 10) of the derivatives showed better efficacy toward the inhibition of *M. tuberculosis* with binding energy of -18.8 kcal/mol as reported in Table 7. Meanwhile, the interaction of the standard drug; isoniazid with the target enzyme was observed with the binding energy -14.6 kcal/mol which was significantly lesser than the binding energy of the ligand (compound 10). This implies that ligand 10 could be used as a structural template to design better hypothetical anti-tubercular drugs with improved activity.

**Table 7:** Molecular docking interactions formed between the prominent ligand and DNA gyrase.

Ligand	Binding energy (BA) Kcal/mol	Hydrogen bond Hydrophobic interaction		
		Amino acid	Bond length (A^o^)	Amino acid
10	-18.8	ARG98 SER118 GLY120 GLY120	3.3701 2.8704 1.9128 3.2821	PRO124, PRO123, VAL97, ASP94, VAL97, ASP122
Isoniazid (Recommended drug)	-14.6	SER279 ALA337	2.2994 2.5295, 2.2466	CYS345, PHE338

#### 3.2.2 Bond type and bond length in the ligand-receptor complex of quiloline derivative

**Figure 5:**
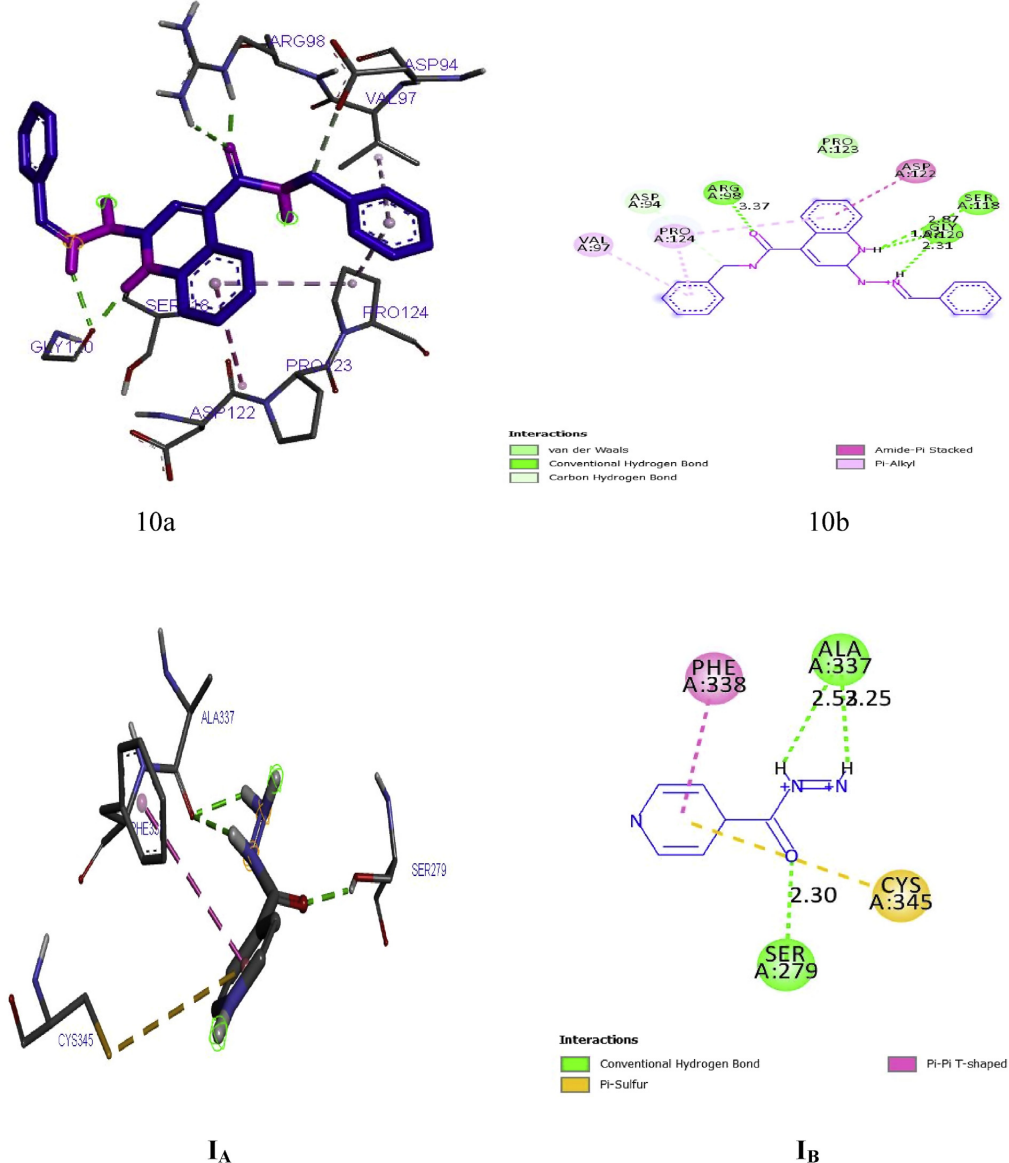
(10a) and (10b) show the 3D and 2D docking interactions between Ligand 10 of quiloline derivatives and DNA gyrase. (IA) and (IB) show the 3D and 2D interactions between Isoniazid and DNA gyrase.

**Figure 6:**
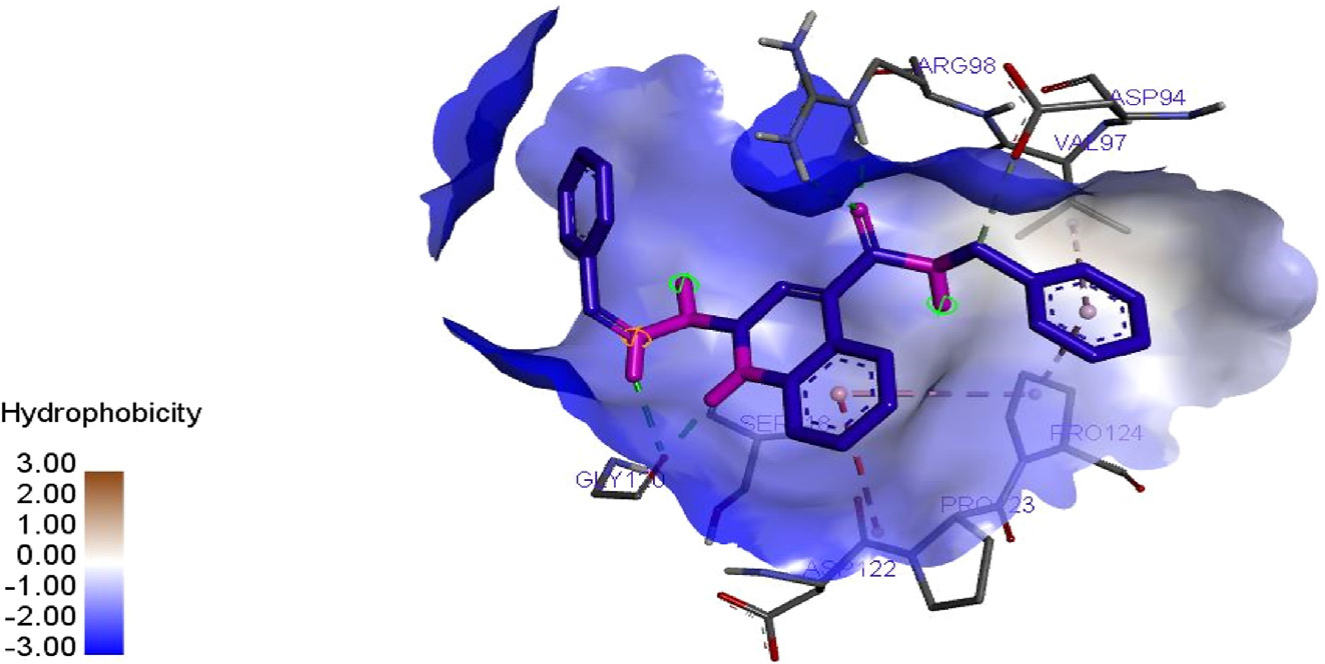
Ligand-receptor hydrophobic interactions between ligand 10 of quinoline derivatives and DNA gyrase.

**Figure 7:**
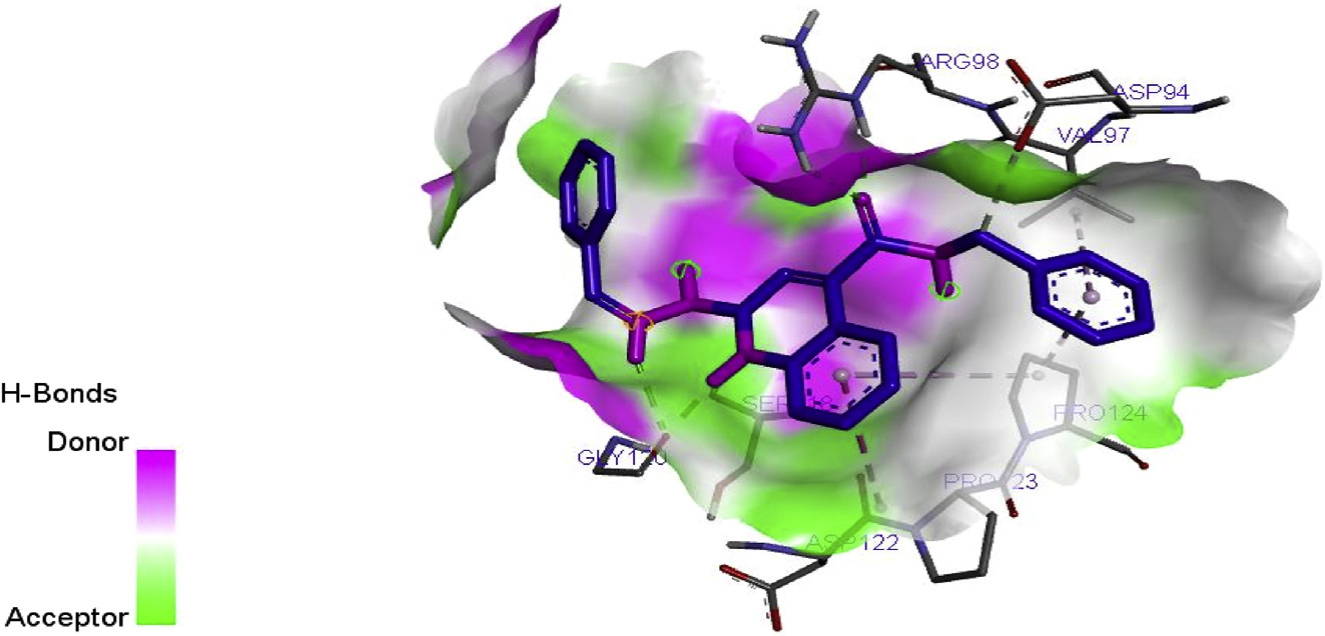
Ligand-receptor H-bond interactions between ligand 10 of quinoline derivatives and DNA gyrase.

The prominent ligand (compound 10) with best binding affinity was s visualized using a discovery studio visualizer software version 2017. The binding interaction in 3-Dimension and 2-Dimension of ligand 10 is represented in Figure 5. Four hydrogen bonding interactions were observed in with this ligand. The amino acid; ARG98, SER118, GLY120 and GLY120 are the main binding site through which the target enzyme bonded with the Ligand via the hydrogen bond length; 3.3701, 2.8704, 1.9128 and 3.2821˚A. The C=O of the quiloline (ligand 10) acts as hydrogen acceptor and formed one H-bond with ARG98 of the enzyme (DNA gyrase). The N–*H group* (hydropyridine) of the quiloline (ligand 10) acts as hydrogen donor and formed two H-bonds with GLY120 of the target. Furthermore, the N–H group (hydrazine) of the quiloline (ligand 10) also acts as hydrogen donor and formed H-bond with SER118 of the target enzyme. More also, hydrophobic interactions were overserved with PRO124, PRO123, VAL97, ASP94, VAL97, ASP122 of the binding site of enzyme as presented in Figure 6. Therefore, the hydrophobic interactions and the H-bonds formation offer a significant evidence to proof that ligand 10 among its co-ligand has the highest efficiency against DNA gyrase receptor. Illuminations of hydrogen acceptor-donor region is shown in Figure 7.

#### 3.2.3 Bond type and bond length in the ligand-receptor complex of recommended drug

The binding interaction in 3-Dimension and 2-Dimension of the target enzyme with the commended drug ‘‘isoniazid’’ is represented in Figure 5. The amino acid; SER279 and ALA337 and ALA337 are the main binding site through which the target enzyme bonded with Isoniazid via the hydrogen bond length; 2.52954, 2.29943 and 2.24657˚A. Meanwhile, the amino acid; CYS345 and PHE338 are the main binding site through which the target enzyme bonded with Isoniazid via the hydrophobic interactions. Based on the observations, increase in number of hydrogen bonds in ligand 10 of quinoline derivatives provide a concrete evidence to support the claim that ligand 10 binds efficiently with the binding pocket of the receptor when compared to the commended drug ‘‘isoniazid’’.

## 4 Conclusion

Quinoline derivatives was study using a theoretical method to select molecular descriptors to relate the structure of the derivatives against *M. tuberculosis*. The validation assessment confirmed that the model is substantial and reliable. Molecular descriptors; AATS7s, VE2_Dzi, SpMin7-Bhe and RDF110i from the results have shown to be prominent descriptor needed to predict the biological activities of the studied compound. Furthermore, docking study indicates that compounds 10 of the derivatives with promising biological activity have the utmost binding energy of -18.8 kcal/mol compared to the commended drugs; Isoniazid -14.6 kcal/mol. The presumption of this research aid the medicinal chemists and pharmacist to design and synthesis a novel drug candidate against the tuberculosis. Moreover, in-*vitro* and in-*vivo* test could be carried out to validate the computational results.

## Declarations

### Author contribution statement

Shola Elijah Adeniji: Conceived and designed the experiments; Performed the experiments; Analyzed and interpreted the data; Contributed reagents, materials, analysis tools or data; Wrote the paper.

Gideon Adamu Shallangwa: Conceived and designed the experiments; Analyzed and interpreted the data.

David Ebuka Arthur: Conceived and designed the experiments; Contributed reagents, materials, analysis tools or data; Wrote the paper.

Mustapha Abdullahi: Conceived and designed the experiments; Contributed reagents, materials, analysis tools or data.

Mahmoud A. Y: Performed the experiments; Wrote the paper.

Abdurrashid Haruna: Performed the experiments; Analyzed and interpreted the data.

### Funding statement

This research did not receive any specific grant from funding agencies in the public, commercial, or not-for-profit sectors.

### Competing interest statement

The authors declare no conflict of interest.

### Additional information

No additional information is available for this paper.

## Appendix A Supplementary data

EMS Table S1_V2 EMS Table S1_V2
