# Supplementary material for: Quantum modelling and molecular docking evaluation of some selected quinoline derivatives as anti-tubercular agents
Source: Heliyon. 2020 Mar 31;6(3):e03639. doi: 10.1016/j.heliyon.2020.e03639 (PMC7114754; doi:10.1016/j.heliyon.2020.e03639)
Supplement: EMS Table S1_V2 [file mmc1.docx]

**Table S1**. Molecular structures of inhibitory compounds and their derivatives as anti-tubercular agents.

| S/N | Molecular structure | Observed Activity (%) | Observed Activity  (pA) | Calculated Activity  (pA) | Residual |
| --- | --- | --- | --- | --- | --- |
| 1^t^ |   (E)-2-(2-(4-methoxybenzylidene)hydrazinyl)-N-phenylquinoline-4-carboxamide | 99 | 9.4858 | 9.7207 | -0.2349 |
| 2 |   (E)-2-(2-(4-methoxybenzylidene)hydrazinyl)-N-phenylquinoline-4-carboxamide | 14 | 6.9651 | 6.8856 | 0.0795 |
| 3 ^t^ |   (E)-N-benzyl-2-(2-(pyridin-3-ylmethylene)hydrazinyl)quinoline-4-carboxamide | 23 | 7.2487 | 6.4992 | 0.7495 |
| 4 |   (E)-N-benzyl-2-(2-(furan-2-ylmethylene)hydrazinyl)quinoline-4-carboxamide | 20 | 7.1586 | 6.9618 | 0.1968 |
| 5 ^t^ |   (E)-N-benzyl-2-(2-(thiophen-2-ylmethylene)hydrazinyl)quinoline-4-carboxamide | 30 | 9.4639 | 9.7549 | -0.2910 |
| 6 |   (E)-2-(2-(anthracen-9-ylmethylene)hydrazinyl)-N-benzylquinoline-4-carboxamide | 20 | 6.9432 | 6.9198 | 0.0234 |
| 7 ^t^ |   (E)-N-benzyl-2-(2-((4-methoxynaphthalen-1-yl)methylene)hydrazinyl)quinoline-4-carboxamide | 16 | 7.2268 | 6.5334 | 0.6934 |
| 8 ^t^ |   (E)-N-benzyl-2-(2-(2-methylpropylidene)hydrazinyl)quinoline-4-carboxamide | 42 | 7.1367 | 6.9960 | 0.1407 |
| 9 ^t^ |   (E)-N-benzyl-2-(2-propylidenehydrazinyl)quinoline-4-carboxamide | 27 | 7.3893 | 7.1755 | 0.2138 |
| 10 |   (E)-N-benzyl-2-(2-benzylidenehydrazinyl)quinoline-4-carboxamide | 99 | 7.2498 | 7.0087 | 0.2411 |
| 11 |   (E)-N-benzyl-2-(2-(4-methoxybenzylidene)hydrazinyl)quinoline-4-carboxamide | 21 | 7.1132 | 7.7017 | -0.5885 |
| 12 |   (E)-N-(5-phenylpentyl)-2-(2-(pyridin-4-ylmethylene)hydrazinyl)quinoline-4-carboxamide | 30 | 7.5695 | 7.7356 | -0.1661 |
| 13 |   (E)-2-(2-(furan-2-ylmethylene)hydrazinyl)-N-(5-phenylpentyl)quinoline-4-carboxamide | 15 | 7.2598 | 6.5187 | 0.7411 |
| 14 |   (E)-N-(5-phenylpentyl)-2-(2-(thiophen-2-ylmethylene)hydrazinyl)quinoline-4-carboxamide | 21 | 9.575 | 9.6508 | -0.0758 |
| 15 ^t^ |   (Z)-2-(2-(anthracen-9-ylmethylene)hydrazinyl)-N-(5-phenylpentyl)quinoline-4-carboxamide | 23 | 7.229 | 7.9095 | -0.6805 |
| 16 |   (E)-2-(2-((4-methoxynaphthalen-1-yl)methylene)hydrazinyl)-N-(5-phenylpentyl)quinoline-4-carboxamide | 40 | 7.4432 | 7.4348 | 0.0084 |
| 17 |   (E)-2-(2-(2-methylpropylidene)hydrazinyl)-N-(5-phenylpentyl)quinoline-4-carboxamide | 42 | 7.0467 | 7.1958 | -0.1491 |
| 18 |   (E)-2-(2-benzylidenehydrazinyl)-N-(5-phenylpentyl)quinoline-4-carboxamide | 21 | 7.2407 | 7.2472 | -0.0065 |
| 19 |   (E)-2-(2-(4-methoxybenzylidene)hydrazinyl)-N-(5-phenylpentyl)quinoline-4-carboxamide | 40 | 7.3751 | 7.6971 | -0.3220 |
| 20 |   (E)-(2-(2-(4-methoxybenzylidene)hydrazinyl)quinolin-4-yl)(morpholino)methanone | 10 | 7.7072 | 7.3417 | 0.3655 |
| 21 |   (E)-(4-methylpiperazin-1-yl)(2-(2-(pyridin-4-ylmethylene)hydrazinyl)quinolin-4-yl)methanone | 28 | 7.6348 | 7.2968 | 0.3380 |
| 22 |   (E)-(2-(2-(furan-2-ylmethylene)hydrazinyl)quinolin-4-yl)(4-methylpiperazin-1-yl)methanone | 21 | 6.2348 | 6.3486 | -0.1138 |
| 23 ^a^ |   (E)-(2-(2-((4-methoxynaphthalen-1-yl)methylene)hydrazinyl)quinolin-4-yl)(4-methylpiperazin-1-yl)methanone | 18 | 7.663 | 7.7607 | -0.0977 |
| 24 |   (E)-(4-methylpiperazin-1-yl)(2-(2-(2-methylpropylidene)hydrazinyl)quinolin-4-yl)methanone | 52 | 6.8074 | 6.8325 | -0.0251 |
| 25 |   (E)-(2-(2-benzylidenehydrazinyl)quinolin-4-yl)(4-methylpiperazin-1-yl)methanone | 9 | 7.3333 | 7.3807 | -0.0474 |
| 26 |   (E)-(2-(2-(4-methoxybenzylidene)hydrazinyl)quinolin-4-yl)(4-methylpiperazin-1-yl)methanone | 30 | 7.1551 | 7.4150 | -0.2599 |
| 27 |   (E)-N-phenyl-2-(2-(thiophen-2-ylmethylene)hydrazinyl)quinoline-4-carboxamide | 26 | 7.1682 | 7.5235 | -0.3553 |
